# Nomogram based on CT imaging and clinical data to predict the efficacy of PD-1 inhibitors combined with chemotherapy in advanced gastric cancer

**DOI:** 10.3389/fimmu.2025.1504387

**Published:** 2025-03-31

**Authors:** Yinchao Ma, Zhipeng Wang, Chenyang Qiu, Mengjun Xiao, Shuzhen Wu, Kun Han, Hui Xu, Haiyan Wang

**Affiliations:** ^1^ Department of Radiology, Shandong Provincial Hospital Affiliated to Shandong First Medical University, Jinan, Shandong, China; ^2^ School of Radiology, Shandong First Medical University, Taian, Shandong, China; ^3^ Department of General Education, Shandong First Medical University, Jinan, Shandong, China

**Keywords:** nomogram, gastric cancer, computed tomography, programmed cell death 1 inhibitors, chemo-immunotherapy

## Abstract

**Background:**

PD-1 inhibitors, in combination with chemotherapy, have become the first-line treatment option for patients with advanced metastatic gastric cancer. However, some patients still do not benefit from this treatment, highlighting an urgent need for simple and reliable markers to predict the efficacy of immunotherapy.

**Methods:**

Immunotherapy efficacy was evaluated using RECIST 1.1 and categorized into complete remission (CR), partial remission (PR), stable disease (SD), and disease progression (PD). Patients with CR, PR, and SD were classified as non-PD responders, while PD patients were categorized as PD responders. Clinical characteristics and CT imaging features of gastric cancer patients from two centers, before receiving PD-1 inhibitor combination chemotherapy, were retrospectively analyzed. A univariate logistic regression analysis was performed for each variable, and separate models for clinical and imaging characteristics, as well as a nomogram, were developed. Area under the curve (AUC), accuracy, sensitivity, specificity, and decision curve analysis (DCA) were used to evaluate all models.

**Results:**

Data from 272 patients (non-PD responders = 206, PD responders = 66) from Center 1 were collected for this study. Data from 76 patients (non-PD responders = 54, PD responders = 22) from Center 2 were used as an external validation cohort to verify the robustness of the models. We developed a clinical model, an imaging features model, and a nomogram. The nomogram, combining clinical and imaging features, demonstrated superior performance with an AUC of 0.904 (95% CI: 0.862–0.947) in the training set and an AUC of 0.801 (95% CI: 0.683-0.918) in the validation set, with sensitivity, specificity, and accuracy of 0.889, 0.682, and 0.829, respectively. Calibration curves further confirmed the agreement between actual results and predictions.

**Conclusions:**

A nomogram combining clinical features and CT imaging features before treatment was developed, which can effectively and simply predict the efficacy response of advanced gastric cancer patients treated with PD-1 inhibitors combined with chemotherapy. This tool can aid in optimizing treatment strategies in clinical practice.

## 1 Introduction

Gastric cancer ranks among the top five cancers worldwide in terms of incidence and mortality (1), and remains a significant threat to human health. Due to its atypical early symptoms and rapid progression, most patients are diagnosed at advanced stages (2). Recent advancements in immunotherapy have transformed cancer treatment, particularly with the introduction of immune checkpoint inhibitors (ICIs), such as anti-programmed cell death-1 (PD-1) and anti-programmed cell death ligand-1 (PD-L1) inhibitors. These agents have made significant strides in treating various malignancies, including advanced gastric cancer (AGC) (3). Anti-PD-1 inhibitors have been officially approved for clinical use in multiple cancers (4), and in recent years, chemotherapy combined with immunotherapy has become a standard regimen for many patients with advanced gastric cancer in clinical practice.

Although PD-1 inhibitors combined with chemotherapy have demonstrated improved efficacy compared to chemotherapy alone in some patients with advanced gastric cancer (5), not all patients benefit from this approach in clinical practice (6). Some patients continue to experience disease progression, immune-related adverse events (7), and potentially excessive medical costs. Therefore, there is an urgent need for simple and reliable predictors of the efficacy of PD-1 inhibitors combined with chemotherapy to identify patients who are likely to benefit.

Current research predominantly focuses on biomarkers such as PD-L1 (programmed death ligand 1) expression, tumor mutational burden (TMB), microsatellite instability-high (MSI-H), and mismatch repair (MMR) status to predict the efficacy of immunotherapy in patients with advanced gastric cancer (8–11). However, many of these biomarker tests require invasive and costly biopsy sampling. In contrast, clinical features such as laboratory tests and imaging findings are more readily accessible and non-invasive. Tumor heterogeneity can complicate the assessment of overall tumor characteristics from biopsy specimens, while imaging tests can provide a comprehensive view of tumor characteristics. Wang et al. (12) demonstrated that changes in certain CT imaging features were associated with the degree of pathological remission in gastric cancer patients undergoing chemotherapy. Additionally, previous studies have explored the relationship between peripheral blood markers or innovative markers derived from them and the prognosis of gastric cancer patients undergoing immunotherapy (13, 14). However, most studies have only proposed associations between predictive factors and clinical outcomes without synthesizing multiple factors into a simple and accurate prediction model.

To more conveniently and accurately assess the efficacy of PD-1 inhibitor combination chemotherapy for gastric cancer, we retrospectively collected clinical characteristics and CT imaging features before treatment from patients with advanced gastric cancer treated with anti-PD-1 inhibitor combination chemotherapy. Our goal is to develop simple and effective predictive models to help identify patients who are likely to benefit from this treatment.

## 2 Materials and methods

### 2.1 Patient inclusion and exclusion criteria

The study was approved by the Ethics Committee of Shandong Provincial Hospital (Ethics number: NSFC: NO.2022-402). Informed consent was waived due to the retrospective nature of the study.

**Figure 1:**
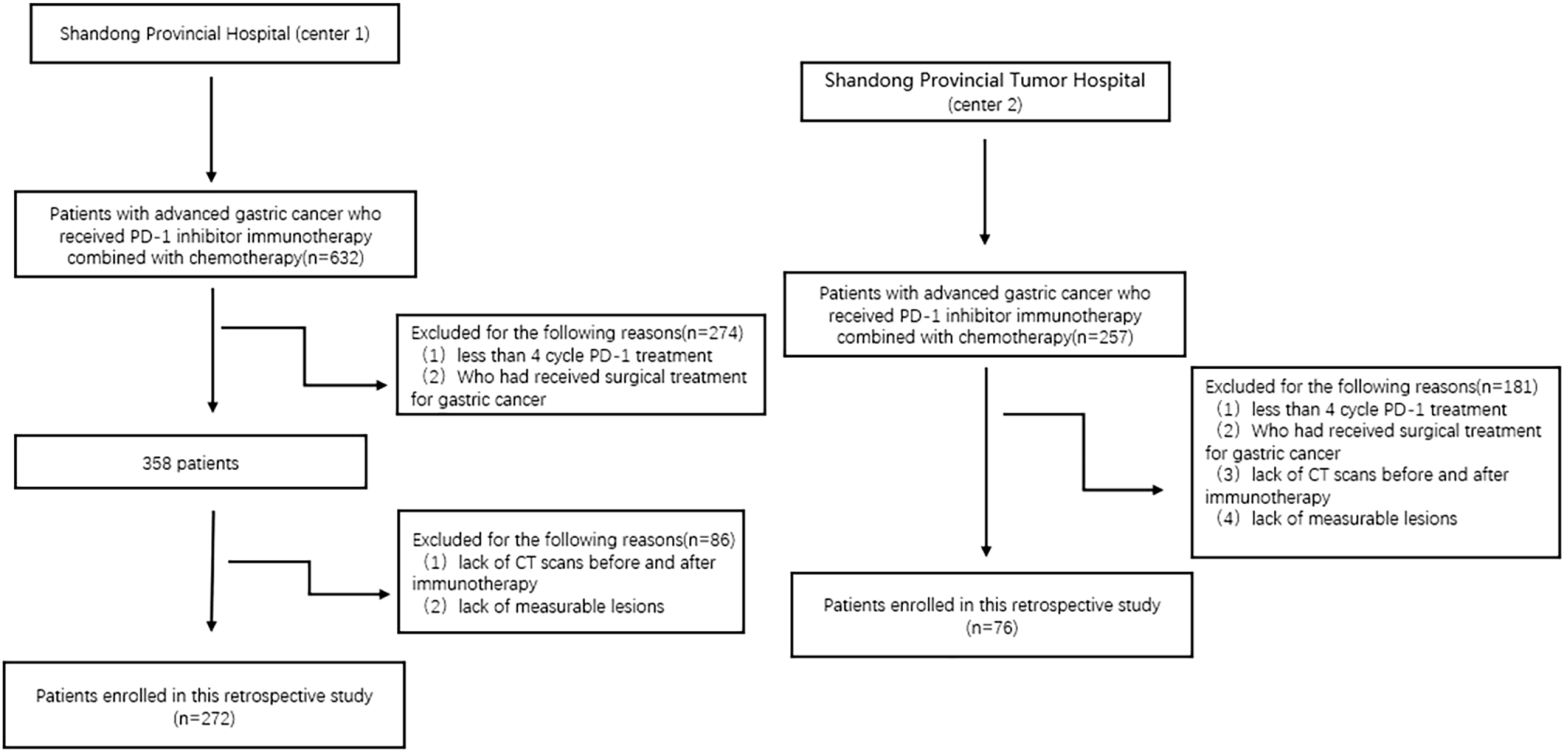
Flowchart of participant selection.

We retrospectively reviewed patients with advanced gastric cancer who were treated with anti-PD-1 inhibitors combined with chemotherapy from June 2019 to June 2023 at Shandong Provincial Hospital of Shandong First Medical University (Center 1) and from June 2019 to June 2023 at Shandong Cancer Hospital (Center 2). The inclusion criteria were as follows: (1) Pathologically confirmed gastric cancer. (2) Receipt of at least four cycles of anti-PD-1 inhibitor combined with chemotherapy. (3) Presence of at least one evaluable tumor lesion meeting the Response Evaluation Criteria in Solid Tumors (RECIST 1.1). (4) Availability of complete CT imaging before and after anti-PD-1 inhibitor combination chemotherapy. Exclusion criteria included: (1) Prior or concurrent surgical treatment of the stomach during anti-PD-1 inhibitor combination chemotherapy. (2) Inadequate gastric distension preventing accurate measurement of the lesion. **Figure 1** shows the participant selection flowchart.

### 2.2 Treatment regimens and outcome assessment

All patients received first-line standard chemotherapy in conjunction with PD-1 inhibitors. The PD-1 inhibitors used in the study included camrelizumab, sintilimab, nivolumab, tislelizumab, and pembrolizumab, administered every three weeks. The primary chemotherapy regimens included SOX (S-1 + oxaliplatin), XELOX (capecitabine + oxaliplatin), and FOLFOX (leucovorin, fluorouracil, and oxaliplatin). The specific chemotherapy regimen was chosen based on the patient’s clinical condition and preference. Tumor response was evaluated approximately four weeks after the completion of four cycles of PD-1 inhibitor combination chemotherapy. The assessment was conducted by radiologists using computed tomography (CT), following the Response Evaluation Criteria in Solid Tumors (RECIST) version 1.1. The response outcomes were categorized as complete remission (CR), partial remission (PR), stable disease (SD), or disease progression (PD). Based on these outcomes, patients were grouped as either non-PD responders (CR, PR, or SD) or PD responders.

### 2.3 Data collection

In this study, clinical characteristics of patients within two weeks prior to their first PD-1 inhibitor combined chemotherapy were obtained through the clinical electronic medical record system. The clinical characteristics include the patient’s gender, age at onset, body mass index (weight[kg]/height [m]²), treatment regimen, presence of distant metastasis, and certain laboratory indicators.

These laboratory indices included alpha-fetoprotein, carcinoembryonic antigen, carbohydrate antigen 125 (CA 125), carbohydrate antigen 19-9 (CA 19-9), carbohydrate antigen 72-4 (CA 72-4), platelet count, and the absolute values of lymphocytes (ALC), monocytes (AMC), and neutrophils (ANC). The inflammation indices included the neutrophil-to-lymphocyte ratio (NLR), calculated as ANC/ALC (absolute neutrophil count [×10^9^/L]/absolute lymphocyte count [×10^9^/L]), the monocyte-to-lymphocyte ratio (MLR), calculated as AMC/ALC (absolute monocyte count [×10^9^/L]/absolute lymphocyte count [×10^9^/L]), and the systemic immune-inflammation index (SII), calculated as (peripheral platelet count [×10^9^/L] × absolute neutrophil count [×10^9^/L])/absolute lymphocyte count [×10^9^/L].

In this study, CT images of patients taken within two weeks before their first PD-1 inhibitor combination chemotherapy and after four cycles of treatment were retrieved from the Picture Archiving and Communication System (PACS). CT imaging characterization was performed by two radiologists with more than three years of experience in abdominal diagnostics. They independently reviewed all CT images using ITK-SNAP (RRID: SCR_017341) (http://www.itksnap.org) (15). The CT images were segmented into lesions using ITK-SNAP, and the lesion areas were outlined as regions of interest (ROI). Disagreements in the assessments were resolved by a senior physician with 30 years of diagnostic radiology experience to reach a consensus. CT signatures included the following characteristics (16, 17): (1) Tumor size (maximum axial diameter and thickness), primary location (based on endoscopic findings: cardia, gastric body, or gastric antrum), and whether the tumor invades the visceral peritoneum; (2) External features: whether the perigastric fat is infiltrated or not and whether the tumor is surrounded by enlarged lymph nodes (defined as regional lymph nodes with a short diameter of 1 cm or more); (3) CT value parameters: The largest cross-section of the tumor was measured in the axial plane, avoiding necrotic and vascular areas, and its CT values were recorded in the plain, arterial, venous, and delayed phases. The final result was the average of measurements from two radiologists. Tumor arterial attenuation (HUA = HU_A_ - HU_U_), venous attenuation (HUV = HU_V_ - HU_U_), and arterial enhancement fraction (AEF) = (HU_A_ - HU_U_)/(HU_V_ - HU_U_) × 100% were also calculated (18) (where HUA represents arterial phase attenuation; HUV represents venous phase attenuation; HU_A_ represents CT values in the arterial phase; HU_V_ represents CT values in the venous phase; and HU_U_ represents CT value of the lesion in the unenhanced phase) (19).

### 2.4 Statistical analysis and nomogram development

Several analytical scales were designed to evaluate clinical and imaging characteristics of the patients. Statistical data were analyzed and visualized using IBM SPSS Statistics (RRID: SCR_019096) (version 25.0; SPSS, Inc., Chicago, IL, USA) and GraphPad Prism (RRID: SCR_002798) (version 9.0; GraphPad, San Diego, CA) for statistical analysis and graphing. Continuous variables with a normal distribution were expressed as mean ± standard deviation, while those not following a normal distribution were expressed as median (interquartile range). Categorical variables were expressed as frequencies and percentages. All statistical tests were two-sided, and a p-value < 0.05 was considered statistically significant.

First, a univariate logistic regression analysis was conducted for each variable. Variables with p < 0.05 were then integrated and modeled using R software (RRID: SCR_001905) (version 4.2.3, https://www.r-project.org/) for clinical and imaging features, respectively. Covariate diagnostics were performed for each model’s variables. Multifactor logistic regression analysis was then conducted by combining indicators with p < 0.05 from both models, and nomograms were developed.

In addition, the area under the curve (AUC) of the Receiver operating characteristic (ROC) of the subjects was plotted in this study to assess the differential diagnostic ability of each model. The DeLong test was used to compare the AUC values between the models. The Hosmer-Lemeshow goodness-of-fit test was used to assess the goodness-of-fit of the nomograms, and calibration curves were plotted to assess the agreement between predicted and actual results. In addition, the performance of all models was assessed by calculating sensitivity, specificity, and accuracy. Finally, clinical decision curve analysis (DCA) was performed in order to understand the patient benefit. The data from center 2 patients were used as an external validation cohort to verify the robustness, generalization ability of each model. There were no statistically significant differences in most of the characteristics of the patients in the two centers (**Table 1**).

**Table 1:** Baseline characteristics of the training cohort and the external validation cohort.

	Training cohort (N=272)			Validation cohort (N=76)		
	PD responders (N=66)	non-PD responders (N=206)	p-value	PD responders (N=22)	non-PD responders (N=54)	p-value
**Age (%)**			0.456			0.819
≤60 years	31 (47.0)	86 (41.7)		10 (45.5)	23 (42.6)	
>60 years	35 (53.0)	120 (58.3)		12 (54.5)	31 (57.4)	
**Gender (%)**			0.224			0.770
Male	45 (68.2)	156 (75.7)		17 (77.3)	40 (74.1)	
Female	21 (31.8)	50 (24.3)		5 (22.7)	14 (25.9)	
**Immunotherapy Regimen (%)**			0.67			0.729
Camrelizumab	36 (54.5)	91 (44.2)		11 (50.0)	25 (46.3)	
Sintilimab	23 (34.8)	88 (42.7)		10 (45.5)	24 (44.4)	
Nivolumab	3 (4.5)	11 (5.3)		0 (0.0)	3 (5.6)	
Tislelizumab	3 (4.5)	10 (4.9)		1 (4.5)	2 (3.7)	
Pembrolizumab	1 (1.5)	6 (2.9)		0	0	
**Tumor location (%)**			0.897			0.847
Cardia	22 (33.3)	74 (35.9)		8 (36.4)	19 (35.2)	
Body	24 (36.4)	69 (33.5)		10 (45.5)	22 (40.7)	
Antrum	20 (30.3)	63 (30.6)		4 (18.2)	13 (24.1)	
**Lymph nodes with a short diameter>1 cm (%)**			0.898			0.77
Absence	19 (28.8)	61 (29.6)		6 (27.3)	13 (24.1)	
Presence	47 (71.2)	145 (70.4)		16 (72.7)	41 (75.9)	
**Obscuration of the perigastric fat space (%)**			0.415			0.935
Absence	9 (13.6)	37 (18.0)		4 (18.2)	12 (22.2)	
Presence	57 (86.4)	169 (82.0)		18 (81.8)	42 (77.8)	
**Serosal invasion (%)**			0.002			0.387
Absence	3 (4.5)	43 (20.9)		1 (4.5)	8 (14.8)	
Presence	63 (95.5)	163 (79.1)		21 (95.5)	46 (85.2)	
**Distant metastasis (%)**			<0.001			0.002
Absence	19 (28.8)	145 (70.4)		4 (18.2)	31 (57.4)	
Presence	47 (71.2)	61 (29.6)		18 (81.8)	23 (42.6)	
**BMI (kg/m^2^)**	23.22 (21.88, 23.73)	23.26 (21.36, 24.61)	0.663	23.23 ± 3.04	23.07 ± 3.28	0.843
**Maximum tumor thickness (cm)**	2.21 (1.50, 2.98)	2.10 (1.63, 2.70)	0.842	2.100 (1.825,2.675)	2.200 (1.500-2.675)	0.566
**Maximum tumor diameter (cm)**	5.55 (4.03, 8.28)	5.850 (4.00, 7.68)	0.718	6.38 ± 2.40	5.99 ± 2.86	0.573
**CT values in the unenhanced phase (HU)**	36.850 (32.33, 39.10)	36.850 (33.10, 40.28)	0.843	31.250 (30.13, 35.40)	32.450 (28.73, 35.20)	0.714
**CT values in the arterial phase (HU)**	59.90 (47.50, 71.38)	67.350 (54.80, 77.15)	0.001	54.050 (49.58, 61.18)	55.300 (49.60, 62.68)	0.457
**CT values in the venous phase (HU)**	77.15 (67.73, 93.23)	77.300 (65.58, 88.85),	0.419	66.750 (61.03, 76.20)	66.450 (60.63, 74.90)	0.909
**CT values in the delayed phase (HU)**	76.80 (67.10, 87.60)	76.050 (67.78, 87.83)	0.883	63.950 (60.50, 73.48)	69.950 (64.18, 77.90)	0.159
**Tumor arterial attenuation (HU)**	21.55 (14.30, 31.48)	30.100 (21.33, 37.98)	<0.001	20.350 (14.35, 28.80)	23.100 (18.53, 29.88)	0.186
**Tumor venous attenuation on portal phase (HU)**	40.25 (32.33, 55.00)	39.650 (31.45, 50.98)	0.533	35.200 (29.00, 45.25)	36.600 (27.13, 44.20)	0.766
**AEF (%)**	55.12 (45.66, 63.75)	71.700 (57.98, 83.61)	<0.001	62.10 (51.75, 68.35)	70.740 (58.68, 75.28)	0.004
**CEA (ng/ml)**	7.96 (2.63, 16.85)	3.98 (1.96, 15.10)	0.037	13.77 (3.91, 48.63)	4.66 (2.00, 34.34)	0.162
**AFP (ng/ml)**	3.00 (1.90, 11.08)	2.50 (1.60, 6.80)	0.095	3.68 (2.33, 18.42)	3.67 (2.11, 9.26)	0.532
**CA125 (U/ml)**	25.17 (12.15, 77.75)	14.40 (8.87, 31.70)	0.001	51.79 (20.88, 187.25)	33.21 (18.75, 79.28)	0.236
**CA199 (U/ml)**	22.34 (12.15, 83.45)	16.95 (10.25, 43.75)	0.146	20.25 (16.60, 126.68)	22.10 (6.35, 110.13)	0.287
**CA724 (U/ml)**	14.90 (4.00, 140.50)	5.66 (2.30, 21.80)	0.001	16.20 (9.70, 32.33)	8.10 (3.53, 18.23)	0.052
**PLT (10^9^/L)**	223.00 (150.75, 276.75)	255.500 (199.00, 300.50)	0.037	259.27 ± 87.60	244.30 ± 91.64	0.515
**lymphocyte (10^9^/L)**	1.26 (0.98, 1.71)	1.525 (1.20, 1.86)	0.003	1.29 (1.09, 1.71)	1.34 (1.12, 1.78)	0.433
Neutrophilcount **(10^9^/L)**	3.58 (2.67, 4.90)	3.26 (2.43, 4.34)	0.118	3.22 (2.81, 4.60)	3.77 (2.79, 4.94)	0.397
Monocyte **(10^9^/L)**	0.50 (0.35, 0.65)	0.47 (0.36, 0.59)	0.72	0.51 (0.41, 0.68)	0.54 (0.42, 0.69)	0.868
SII	541.76 (350.56, 1044.94)	517.41 (323.15, 893.94)	0.302	657.72 (411.83, 970.80)	666.99 (361.34, 1008.44)	0.805
MLR	0.39 (0.29, 0.53)	0.32 (0.24, 0.43)	0.002	0.36 (0.30, 0.53)	0.41 (0.28, 0.50)	0.779
NLR	2.65 (1.86, 4.56)	2.05 (1.53, 3.15)	0.002	2.69 (2.08,3.98)	2.79 (2.09,4.01)	0.963

## 3 Results

### 3.1 Patients

A total of 272 patients (206 non-PD responders and 66 PD responders) from Center 1 and 76 patients (54 non-PD responders and 22 PD responders) from Center 2 were included in this study. Patients from center 1 were used as a training set to build the model, and patients from center 2 were used as an external validation cohort to test the robustness of the model.

### 3.2 Clinical features

Each enrolled patient was classified as either a PD responder or non-responder based on treatment efficacy after receiving four cycles of PD-1 inhibitor combination chemotherapy. In the training cohort, no statistically significant differences were observed between the PD responder and non-PD responder groups in terms of age, gender, and body mass index (BMI) (p > 0.05). However, the presence of distant metastases was significantly different between the two groups (p < 0.05). Laboratory tests revealed no significant differences between the PD responder and non-PD responder groups in alpha-fetoprotein, carcinoembryonic antigen, carbohydrate antigen 125 (CA 125), carbohydrate antigen 19-9 (CA 19-9), platelet count, or absolute monocyte count (AMC). Conversely, significant differences were observed in carbohydrate antigen 72-4 (CA 72-4), absolute neutrophil count (ANC), absolute lymphocyte count (ALC), neutrophil-to-lymphocyte ratio (NLR), monocyte-to-lymphocyte ratio (MLR), and systemic immune-inflammation index (SII) (p < 0.05). Univariate and multivariate logistic regression analyses of clinical characteristics are presented in **Table 2**.

**Table 2:** Univariate and multivariate logistic regression analysis of clinical features.

Variable	Univariate analysis		Multivariate analysis	
	OR (95%CI)	P-value	OR (95%CI)	P-value
**Age (year)**	1.24 (0.71-2.16)	0.456		
**Gender**	0.69 (0.37-1.26)	0.226		
**BMI (kg/m^2^)**	1.03 (0.93-1.14)	0.569		
**Distant metastasis**	0.17 (0.09-0.31)	<0.001	0.22 (0.11-0.44)	<0.001
**AFP (ng/ml)**	1.00 (1.00-1.00)	0.161		
**CEA (ng/ml)**	1.00 (1.00-1.00)	0.352		
**CA125 (U/ml)**	1.00 (1.00-1.00)	0.277		
**CA199 (U/ml)**	1.00 (1.00-1.00)	0.265		
**CA724 (U/ml)**	0.99 (0.99-0.99)	<0.001	0.99 (0.98-0.99)	<0.001
**PLT (10^9^/L)**	1.00 (1.00-1.01)	0.06		
**lymphocyte (10^9^/L)**	2.47 (1.33-4.59),	0.004	0.73 (0.18-2.92)	0.659
**Monocyte (10^9^/L)**	0.62 (0.16-2.47)	0.501		
**Neutrophilcount (10^9^/L)**	0.86 (0.75-0.99)	0.035	1.26 (0.78-2.04)	0.353
**NLR**	0.71 (0.60-0.85)	<0.001	0.43 (0.23-0.83)	0.012
**MLR**	0.06 (0.01-0.35)	0.002	1.72 (0.13-22.53)	0.681
**SII**	1.00 (1.00-1.00)	0.046	1.00 (1.00-1.00)	0.057

### 3.3 Computed tomography imaging features

CT examination showed no statistically significant differences between the two groups regarding tumor location, maximum diameter, maximum thickness, whether the surrounding fat was infiltrated, or the presence of enlarged lymph nodes in the surrounding area (p > 0.05). However, the presence of serosal invasion (involving the visceral peritoneum) showed a statistically significant difference between the PD responder and non-PD responder groups (p < 0.05). There were no statistically significant differences in CT values or tumor venous attenuation values between the plain, venous, and delayed phases (p > 0.05). Notably, CT values, arterial attenuation values, and arterial enhancement fraction (AEF) of tumors in the arterial phase were significantly higher in the non-PD responder group compared to the PD responder group (p < 0.05). Univariate and multivariate logistic regression analyses of CT imaging features are shown in **Table 3**.

**Table 3:** Univariate and multivariate logistic regression analysis of imaging features.

Variable	Univariate analysis		Multivariate analysis	
	OR (95%CI)	P-value	OR (95%CI)	P-value
**Tumor location**	0.97 (0.69-1.36)	0.897		
**Maximum tumor thickness (cm)**	0.86 (0.65-1.13)	0.283		
**Maximum tumor diameter (cm)**	1.00 (0.91-1.11)	0.965		
**Lymph nodes with a short diameter>1 cm**	0.96 (0.52-1.77)	0.898		
**Obscuration of the perigastric fat space**	0.72 (0.33-1.59)	0.416		
**Serosal invasion**	0.18 (0.05-0.60)	0.005	0.12 (0.03-0.43)	0.001
**CT values in the unenhanced phase (HU)**	0.99 (0.95-1.04)	0.790		
**CT values in the arterial phase (HU)**	1.04 (1.02-1.06)	0.001	0.98 (0.92-1.04)	0.548
**CT values in the venous phase (HU)**	0.99 (0.98-1.01)	0.439		
**CT values in the delayed phase (HU)**	1.00 (0.98-1.01)	0.906		
**Tumor Arterial attenuation (HU)**	1.05 (1.03-1.08)	<0.001	1.00 (0.93-1.08)	0.927
**Tumor venous attenuation on portal phase (HU)**	0.99 (0.98-1.01)	0.439		
**AEF (%)**	1.08 (1.06-1.11)	<0.001	1.10 (1.06-1.13)	<0.001

### 3.4 Development of differentiation models for PD and non-PD patients

**Figure 2:**
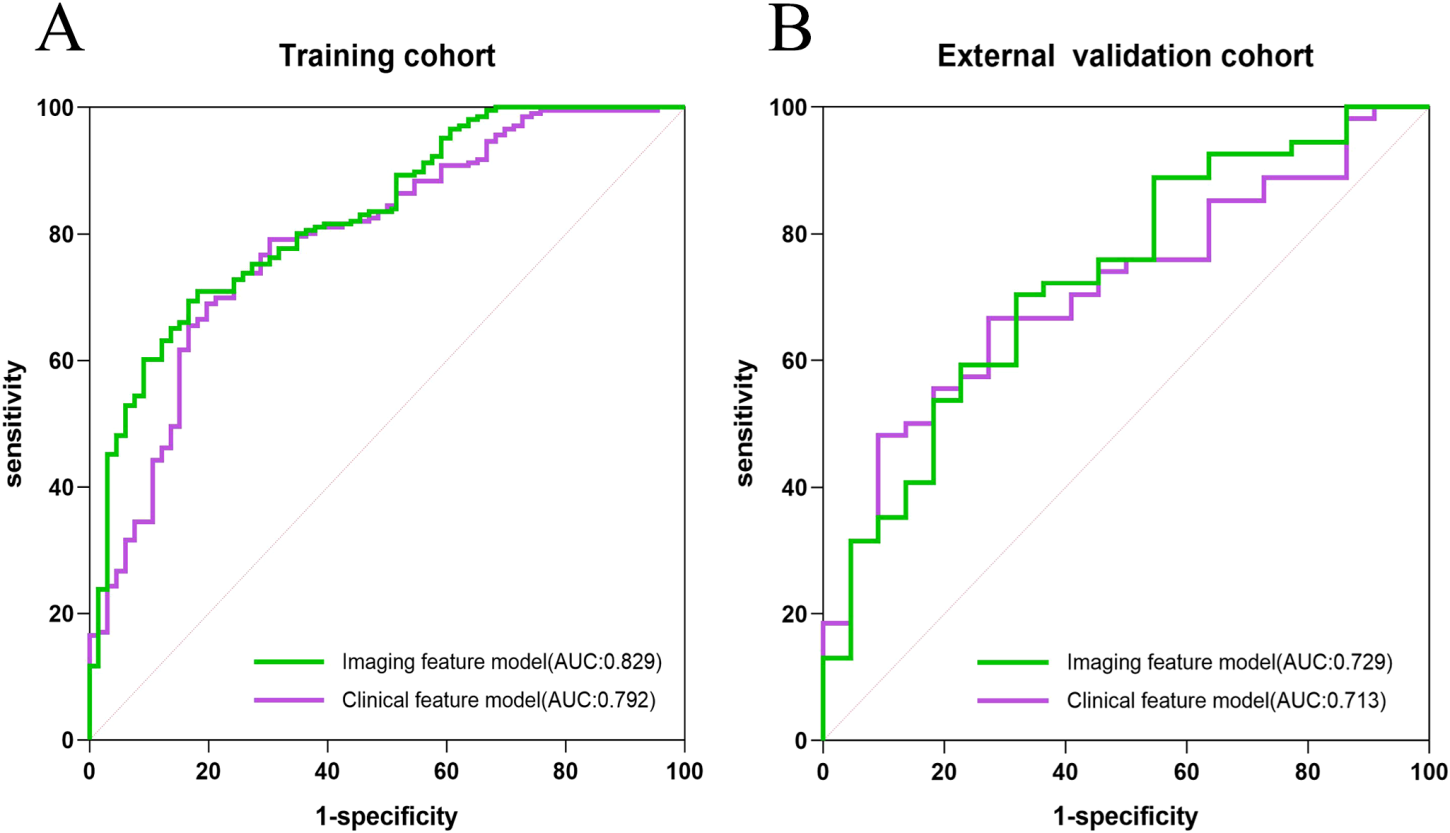
The ROC curves of the clinical model, and imaging features model in the training cohort **(A)** and the external validation cohort **(B)**, respectively.

Comparative analyses of laboratory parameters and imaging features were conducted to develop the most effective model for optimal discrimination. Initially, all indicators were analyzed individually using univariate logistic regression, and those with p < 0.05 were included in subsequent multivariate logistic regression analysis. Clinical and imaging feature models were established separately, with covariate diagnosis performed for each. The clinical feature model included carbohydrate antigen 72-4 (CA 72-4), the neutrophil-to-lymphocyte ratio (NLR), and the presence of distant metastasis. The imaging feature models included the arterial enhancement fraction (AEF) and the presence or absence of visceral peritoneum infiltration. The area under the curve (AUC) for the clinical and imaging feature models in the training cohort were 0.792 and 0.829, respectively, while in the external validation cohort, the AUCs were 0.713 and 0.729, respectively. The ROC curves for the two models are shown in **Figure 2**.

### 3.5 Development and evaluation of the nomogram

**Figure 3:**
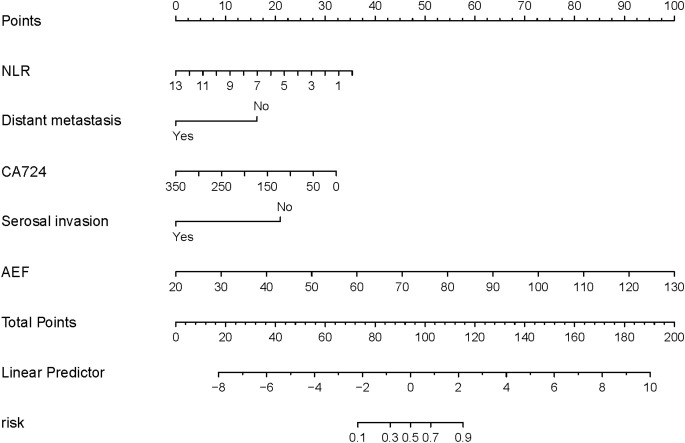
Nomogram based on clinical features and imaging features.

Indicators with p < 0.05 from both the clinical feature model and the imaging feature model were selected to construct the nomogram. These included the neutrophil-to-lymphocyte ratio (NLR), CA 72-4, the presence of distant metastasis, the presence of visceral peritoneum infiltration, and the arterial enhancement fraction (AEF). The nomogram is illustrated in **Figure 3**.

**Figure 4:**
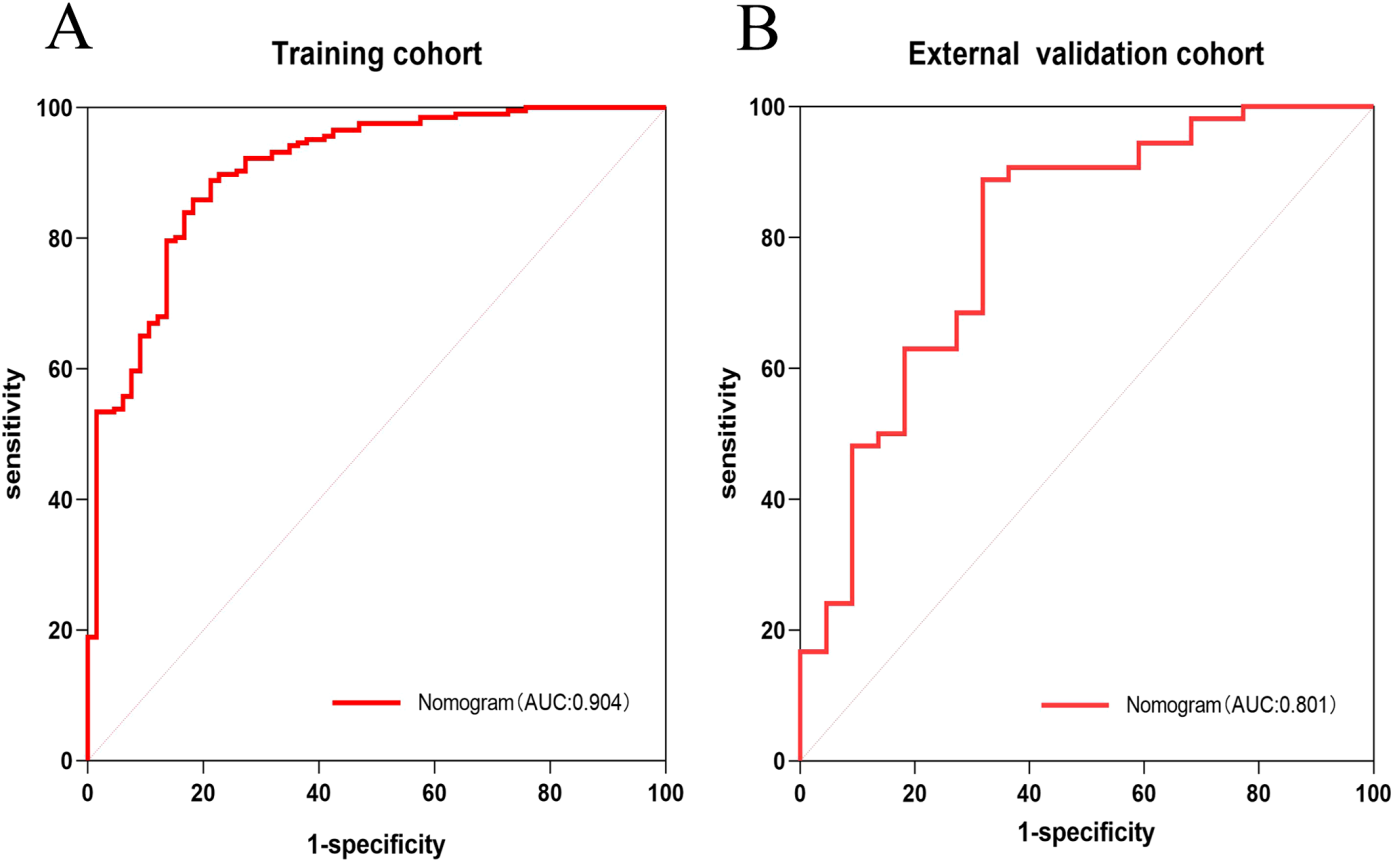
ROC curves of Nomogram in training cohort and external validation cohort. ROC curve of nomogram in the training cohort **(A)**. ROC curve of nomogram in the external validation cohort **(B)**.

**Figure 5:**
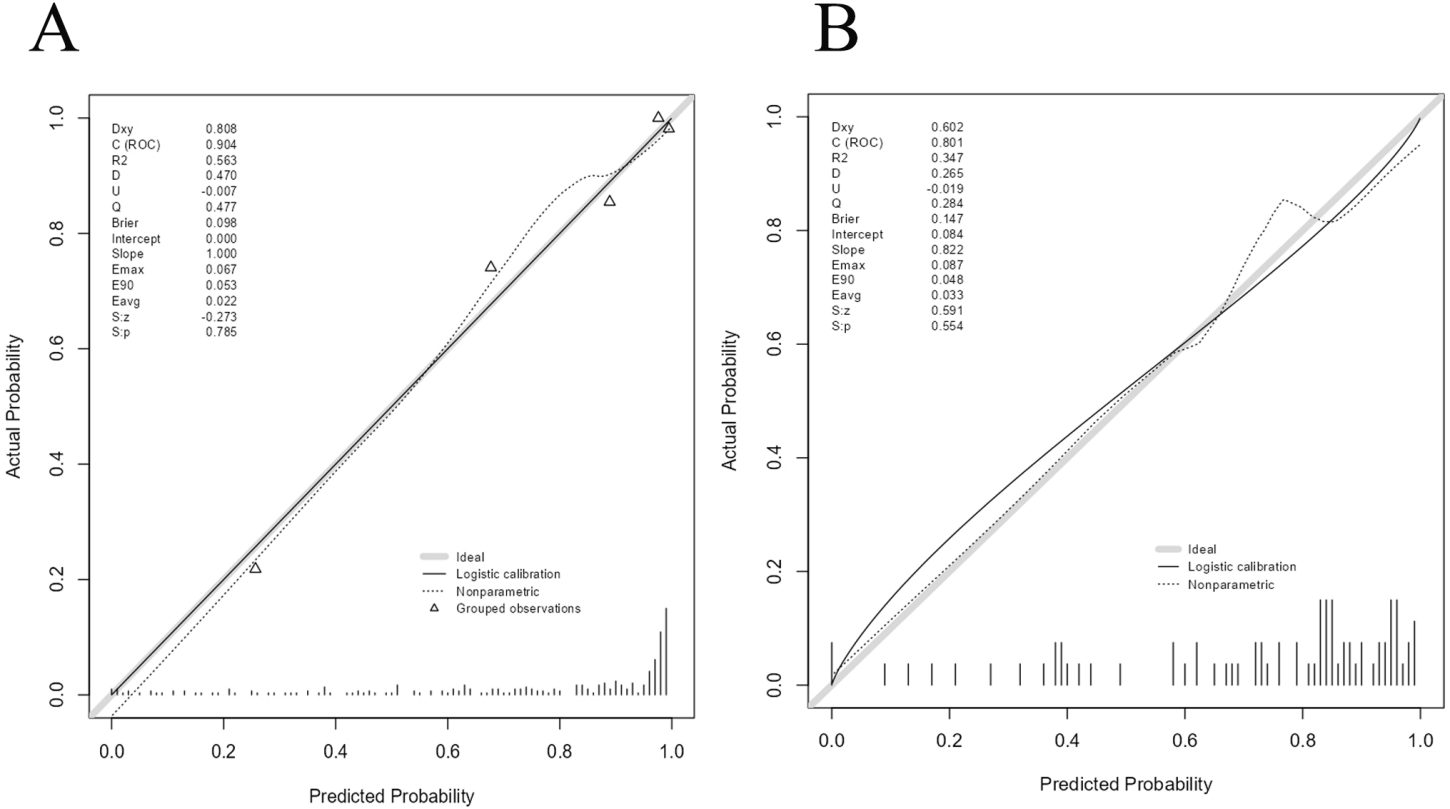
Calibration curves of Nomogram in training cohort and external validation cohort.Calibration curve of nomogram in the training cohort **(A)**. Calibration curve of nomogram in the external validation cohort **(B)**.

**Figure 6:**
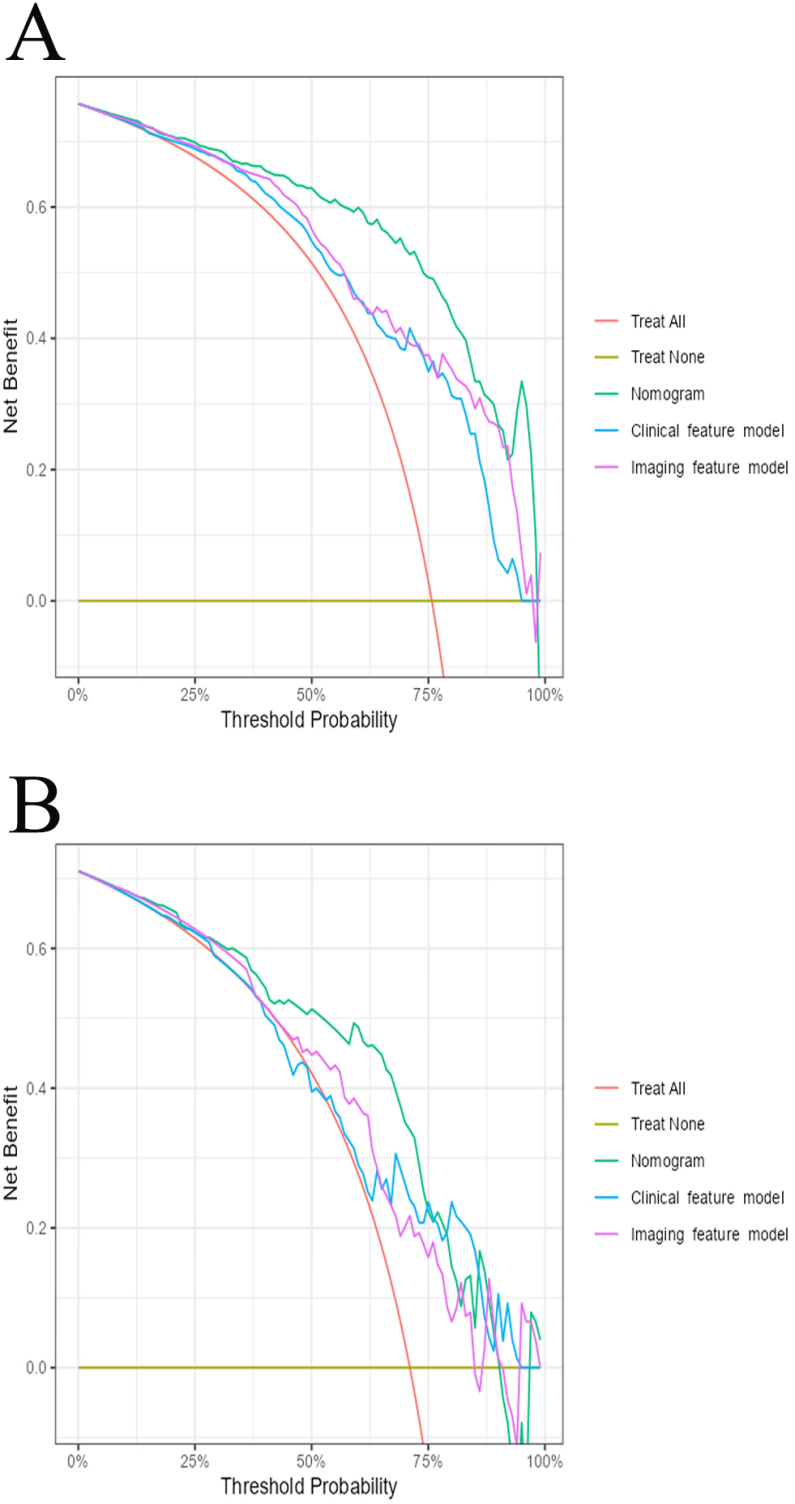
The decision curve analysis for all models in training cohort **(A)** and external validation cohort **(B)**.

The AUC for the nomogram was 0.904 in the training cohort and 0.801 in the external validation cohort (**Figure 4**). The DeLong test indicated a statistically significant difference between the nomogram and both the clinical and imaging feature models in both cohorts (p < 0.05). According to the Hosmer-Lemeshow goodness-of-fit test, the nomogram demonstrated good fit in both the training cohort (p = 0.236) and the external validation cohort (p = 0.191). The nomogram also showed strong agreement with the calibration curves for both cohorts (**Figure 5**). Additionally, decision curve analysis for the clinical feature model, imaging feature model, and nomogram is depicted in **Figure 6**, showing that the nomogram provided the highest net benefit. The accuracy, specificity, and sensitivity of all models are detailed in **Table 4**.

**Table 4:** The performance of models in the training cohort and external validation cohort.

Cohort	Models	AUC	Accuracy	Specificity	Sensitivity
Training cohort	Clinical model	0.792 (95% CI:0.730-0.855)	0.717	0.803	0.689
	Imaging model	0.829 (95% CI:0.775-0.883)	0.728	0.833	0.694
	Nomogram (clinical+imaging)	0.904 (95% CI:0.862-0.947)	0.849	0.818	0.859
External validation cohort	Clinical model	0.713 (95% CI:0.593-0.833)	0.684	0.727	0.667
	Imaging model	0.729 (95% CI:0.604-0.854)	0.697	0.682	0.704
	Nomogram (clinical+imaging)	0.801 (95% CI:0.683-0.918)	0.829	0.682	0.889

## 4 Discussion

In this study, we used clinical and CT imaging features before treatment to predict the early treatment response of gastric cancer patients receiving PD-1 inhibitors combined with chemotherapy. The nomogram that integrated both clinical and imaging features showed the best performance, with AUCs of 0.904 in the training set and 0.801 in the validation set. This performance surpassed both the clinical feature model and imaging feature model alone and slightly outperformed the radiomics model by Liang et al. (20). In a previous study, Li et al. (21) developed a nomogram to predict complete pathological remission in gastric cancer patients undergoing immunotherapy combined with chemotherapy, using tumor diameter, clinical N stage, and the combined positive score (CPS) of PD-L1. In contrast, our nomogram incorporates readily available, non-invasive biomarkers, eliminating the need for immunohistochemical markers like PD-L1 CPS from biopsy samples, thereby simplifying data acquisition while maintaining high predictive accuracy.

Liang et al. (20) previously constructed a model using radiomics-extracted imaging features combined with two clinical risk factors to predict the efficacy of PD-1 inhibitors in gastric cancer patients, achieving an AUC of 0.865 in the training set and 0.778 in the internal validation set. While their model demonstrated strong predictive performance, our nomogram offers a more accessible approach by incorporating standard CT imaging features, simplifying the process of obtaining lesion data. Moreover, our nomogram’s AUC, sensitivity, and specificity are slightly higher than Liang’s model, and we further validated our nomogram externally, supporting its generalizability and robustness.

In this study, we initially developed a clinical characterization model, which achieved an area under the curve (AUC) of 0.792 in the training cohort. However, this model exhibited a lower sensitivity of 0.689, and the decision curve analysis (DCA) indicated a reduced patient benefit. Subsequently, we developed an imaging feature model based on CT imaging characteristics. This model demonstrated superior performance compared to the clinical feature model, with an AUC of 0.829, and improved accuracy and specificity. Nevertheless, the sensitivity of the CT imaging model was comparable to that of the clinical feature model, at 0.694. Finally, we integrated the statistically significant predictors (p < 0.05) from both models to construct a nomogram, which simplified the metrics while enhancing diagnostic efficacy. The nomogram exhibited excellent predictive performance, achieving an AUC of 0.904, surpassing both the CT imaging feature model and the clinical feature model. Additionally, the nomogram demonstrated sensitivity, specificity, and accuracy exceeding 0.80, and showed promising results in external validation with an AUC of 0.806.

We constructed the nomogram based on five relevant predictive factors (p < 0.05) identified from the clinical feature model and imaging feature model. The clinical model included three key factors: neutrophil-to-lymphocyte ratio (NLR), CA724, and the presence of distant metastases. NLR (neutrophils/lymphocytes) has been found to be positively correlated with the density of exhausted CD8 T immune cells in the tumor microenvironment (22), and exhausted CD8 T cells represent a state of progressively diminished T-cell function (23). High levels of serum carbohydrate antigen 72-4 (CA72-4) typically indicate the persistence and proliferation of tumor cells (24), and previous studies have demonstrated that CA72-4 can predict chemotherapy efficacy in gastric cancer patients (25) as well as prognosis following immunosuppressive therapy (26). The presence of tumor metastasis is considered a key factor influencing the effectiveness of PD-1 inhibitor therapy in gastric cancer patients (27). Tumor cells and the tumor immune microenvironment (TME) within metastatic lesions may impact the local immune microenvironment of distant tissues via circulation (28), thus affecting the response to immunotherapy.

Relevant predictors (p < 0.05) from the imaging characterization model used in our nomogram included arterial enhancement fraction (AEF) and the presence or absence of serosal invasion (visceral peritoneal). Our study revealed that tumors with a higher AEF on pre-treatment CT images were more likely to show a better response to treatment in patients with advanced gastric cancer undergoing PD-1 inhibitor combination chemotherapy. This observation may be related to the association between higher AEF values and elevated composite positive score (CPS) for PD-L1 expression (29), with gastric cancer patients who have high CPS scores for PD-L1 potentially deriving greater benefit from immunotherapy (30). Additionally, serosal invasion (visceral peritoneal) is a significant factor affecting the prognosis of gastric cancer patients (31). Such invasion may contribute to the development of an immunosuppressive inflammatory tumor microenvironment through chronic inflammation (32, 33), which can subsequently impact the effectiveness of immunotherapy.

In our study, we developed a straightforward and effective nomogram using the predictors mentioned above, which are easily accessible and non-invasive. In contrast to individual biomarker predictions, our nomogram assists clinicians in forecasting treatment efficacy and crafting personalized treatment strategies. By quantifying each predictor as numerical probabilities, it enables more accurate early-stage predictions of treatment response for gastric cancer patients undergoing PD-1 inhibitor combination chemotherapy.

As with most retrospective studies, our research has several limitations. Firstly, the study population was relatively small, indicating the need for larger, multicenter prospective studies to confirm our findings. Secondly, although CT imaging is a crucial tool for assessing the efficacy of immunotherapy in gastric cancer, discrepancies may arise between CT assessments and pathological findings, which can affect the comprehensiveness and accuracy of the efficacy evaluation. Since this was a retrospective study, most patients did not undergo surgical resection and therefore the degree of their pathological remission could not be determined, further limiting our ability to comprehensively assess treatment efficacy. Therefore, in future studies, we plan to include more cases and use the degree of pathological remission as a criterion for efficacy assessment to enhance the accuracy and comprehensiveness of evaluating immunotherapy efficacy.

## 5 Conclusion

In summary, we developed and validated a nomogram utilizing clinical features before treatment (NLR, CA724, and the presence or absence of distant metastases) alongside CT imaging features before treatment (arterial enhancement fraction and serosal invasion). This nomogram serves as a simple and effective tool for early prediction of therapeutic efficacy in patients with advanced gastric cancer receiving PD-1 inhibitor-combined chemotherapy, thereby aiding clinicians in formulating personalized treatment plans.

## Data Availability

The original contributions presented in the study are included in the article/**Supplementary Material**. Further inquiries can be directed to the corresponding authors.
