# Supplementary material for: Nomogram based on CT imaging and clinical data to predict the efficacy of PD-1 inhibitors combined with chemotherapy in advanced gastric cancer
Source: Front Immunol. 2025 Mar 31;16:1504387. doi: 10.3389/fimmu.2025.1504387 (PMC11994692; doi:10.3389/fimmu.2025.1504387)
Supplement: Supplementary file 1 [file Table1.docx]

Supplementary Material

Nomogram based on CT imaging and clinical data to predict the efficacy of PD-1 inhibitors combined with chemotherapy in advanced gastric cancer

**Yinchao Ma^†^• Zhipeng Wang^†^•Chenyang Qiu • Mengjun Xiao • Shuzhen Wu• Kun Han•**

**Hui Xu*•Haiyan Wang***

*** Correspondence:**

Hui Xu

e-mail: [hxu@sdfmu.edu.cn](mailto:hxu@sdfmu.edu.cn) ；

Haiyan Wang

# [whyott@163.com](mailto:whyott@163.com)

# Supplementary Tables

Supplementary Table 1. CT scan protocols

| \| **Modality** \| **CT** \| \| \| \| \| \| --- \| --- \| --- \| --- \| --- \| --- \| \| **Scanner model** \| SOMATOM Force \| SOMATOM Definition Flash \| SOMATOM Drive \| Aquilion ONE \| Ingenuity CT \| \| **Manufacturer** \| SIEMENS \| SIEMENS \| SIEMENS \| TOSHIBA \| Philips \| \| **Tube voltage (kV)** \| 90 \| 120 \| 120 \| 120 \| 120 \| \| **Tube current** \| 245 \| 256 \| 411 \| 100 \| 240 \| \| **Matrix** \| 512×512 \| 512×512 \| 512×512 \| 512×512 \| 512×512 \| \| **Slice thickness (mm)** \| 1 \| 1 \| 1 \| 1 \| 1 \| \| **Exposure time (ms)** \| 500 \| 500 \| 330 \| 500 \| 415 \|   kV, kilovoltr; mm, millimeter; ms, millisecond |  |  |  |  |
| --- | --- | --- | --- | --- | --- | --- | --- | --- | --- | --- | --- | --- | --- | --- | --- | --- | --- | --- | --- | --- | --- | --- | --- | --- | --- | --- | --- | --- | --- | --- | --- | --- | --- | --- | --- | --- | --- | --- | --- | --- | --- | --- | --- | --- | --- | --- | --- | --- | --- | --- | --- | --- |

**
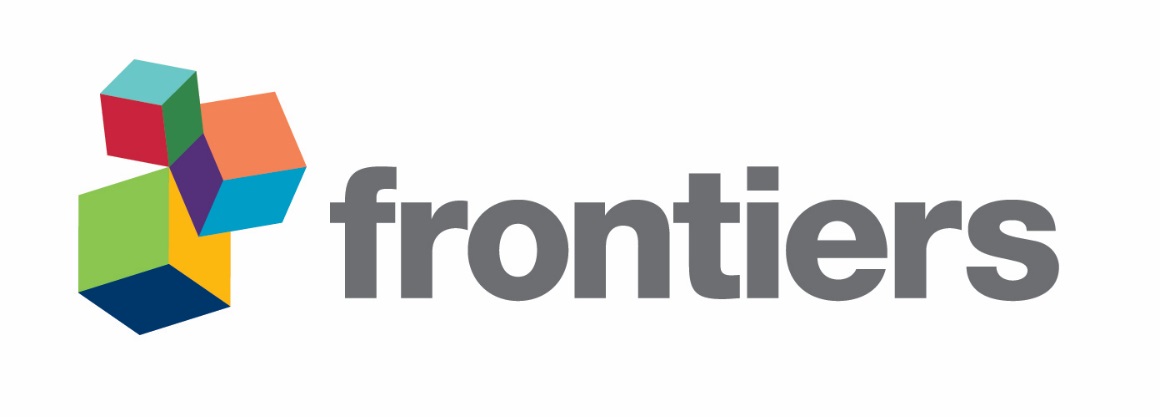
**
